# Knowledge, Attitude and Behavioural Responses Towards Mental Illness Among Pharmacy Students in a Tertiary Teaching Hospital in South India: A Cross-Sectional Study

**DOI:** 10.7759/cureus.72065

**Published:** 2024-10-21

**Authors:** Shreya Haridas, Shilpa Pramoj, Kathleen A Mathew

**Affiliations:** 1 Pharmacy, Amrita Institute of Medical Sciences and Research, Kochi, IND; 2 Psychiatry, Amrita Institute of Medical Sciences and Research, Kochi, IND

**Keywords:** attitudes of mental illness, attitude toward psychiatry, mental health awareness, psychiatry, stigma against mental health issues, stigma in health care

## Abstract

Background

Individuals with mental illness are frequently subjected to discriminatory and stigmatising behaviour in society. Promoting positive attitudes and improving awareness regarding mental illness among health professionals in training are important steps in mitigating stigma towards mental illness. There is a dearth of Indian studies which have looked into these aspects. The observations of the study aimed to assess whether stereotyped views on mental illness pertain among pharmacy students at a tertiary teaching institution in South India.

Objective

The study evaluates three key aspects related to pharmacy students and mental illness: 1) the attitude of pharmacy students towards individuals with mental illness, 2) their knowledge about mental illness and 3) their behavioural response towards mental illness.

Materials and methods

A cross-sectional study was conducted among the students enrolled in Doctor of Pharmacy (PharmD), Bachelor of Pharmacy (BPharm) and Master of Pharmacy (MPharm) programs in a teaching hospital in south India. Three hundred one completed responses were obtained. The study tools included a socio-demographic proforma, Mental Illness: Clinicians’ Attitude Scale (MICA-4), Mental Health Knowledge Schedule (MAKS) and Reported and Intended Behaviour Scale (RIBS).

Results

The mean scores obtained on MICA-4, MAKS and RIBS were 44.52 ± 7.662, 45.3156 ± 7.601 and 13.18 ± 4.884, respectively; 59.8% of the participants considered psychiatric patients as dangerous, and 70.1% of the respondents reported that they were capable of guiding a friend with a mental illness in accessing professional help. Female students had significantly lower MICA-4 scores than males (p = 0.001). First-year students had significantly lower RIBS scores as compared to the students in the second to the sixth years of study (p = 0.004).

Conclusion

Negative attitudes, stigmatising beliefs and behaviour towards mental illness are prevalent among future pharmacists. It is the need of the hour to ensure that mental health-related content and contact-based education aimed at reducing stigma and discrimination receive due emphasis as part of the pharmacy curriculum in the country.

## Introduction

Mental health is defined as a condition of contentment in which a person recognises their capability, can handle the customary stresses of survival, engages in productive and rewarding employment and can provide a beneficial hand to his or her faction [1]. In this galloping world, mental disorders are increasingly being detected and diagnosed. WHO statistics show that one in every eight people, or other words, 970 million people around the world, suffer from mental illnesses. According to the findings of the National Mental Health Survey of 2015-16, 13.6% of the population in India experienced a mental illness during their lifetime. Another striking finding of this survey was that the treatment gap for major mental illnesses ranged between 70% and 92% [2]. This indicates that though effective treatment options are available, many people face major hurdles, including stigma and discrimination, in accessing timely mental healthcare.

Stigma refers to a mark of shame or disrepute that distinguishes a person from other individuals and precludes one from receiving complete social acceptability [3]. Persons suffering from mental illness are often subjected to stigma and segregation in society, even within their families. A community-based study conducted in south India observed that 74.6% of the 445 participants reported stigma towards persons with mental illness [4]. Stigma and discrimination are major factors that escalate the likelihood of treatment avoidance, delay in seeking care, and drop-out from treatment among individuals with mental illness [5].

In a clinical setting, the stigmatising beliefs and discriminatory attitudes of healthcare workers towards patients with mental illness can negatively impact patient care. Health professionals’ misconception that mental disorders are self-inflicted and that they are beyond cure can lead to therapeutic pessimism [6]. Prejudicial behaviours towards people with mental illness can hinder the accessibility of professional help for these patients and affect the quality of mental health care provided to them [7].

In the wake of the rising disease burden of mental illnesses worldwide and a paradigm shift in mental health care systems to include a multi-disciplinary team approach, the role of pharmacists has expanded to much beyond dispensing medications. Students training to be pharmacists would have a crucial part to play in future in the management of mental illness, especially when it comes to medication-related education, which is a determining factor of treatment compliance [8]. This necessitates a need for future pharmacists to address their negative attitude towards mental illness and equip themselves with knowledge about a range of mental health disorders, including being able to identify characteristic symptoms and have an understanding of the treatment options [9].

There is a dearth of Indian studies that have looked into the attitudes, behaviours and levels of knowledge concerning mental illness and associated stigma among pharmacy students in India. Promoting positive attitudes and improving awareness regarding mental illness among these health professionals-in-training will have paramount importance in mitigating public stigma and, ultimately, self-stigma among mentally ill individuals [10]. The current study aims to determine the attitudes and behaviours towards people with mental illness in addition to knowledge about mental illnesses among pharmacy students at a tertiary care teaching institution in South India.

## Materials and methods

Study design and setting

We conducted a cross-sectional study involving the students enrolled in Doctor of Pharmacy (PharmD), Bachelor of Pharmacy (BPharm) and Master of Pharmacy (MPharm) programs at Amrita Institute of Medical Sciences, Kochi, Kerala, India.

Ethics

Approval from the Institutional Ethics Committee clearance was obtained with reference number IEC AIMS-2022-PHARM-348. The study duration was extended over two months (February and March 2023). All students in the abovementioned programs were invited to take part in the study. Participants were required to give their informed consent before starting the study. The data collection form consisting of sociodemographic details (age, gender, course, year of study and family history of psychiatric illness) and the study tools, which included MICA-4, MAKS and RIBS, were distributed to the students. Incomplete responses were excluded from the study.

Sample size

Based on the results of the mean ± SD of attitude (45.6 ± 6.68) of pharmacy students towards people with mental illness using the MICA-4 scale observed from a pilot study conducted with 20 samples and with ±1-unit absolute precision, the minimum sample size came to 171.

Study tools

Mental Illness: Clinicians’ Attitude Scale Version 4 (MICA-4)

This scale was utilised to determine the participants’ attitudes concerning mental illness. Version 4 is used for most health professional groups and students. The scoring is on a Likert-type scale comprising 16 items with six options for each item. A score of one is indicative of strong agreement, while six denotes strong disagreement. The item scores are summed up to obtain a total score in the range of 16 to 96. Higher scores point towards a more negative attitude. Studies on the psychometric properties of this scale have demonstrated adequate reliability (Cronbach's α ≥ 0.7). Studies have demonstrated adequate face validity and convergent validity for this scale. The scale was also found to be moderately correlated with RIBS in previous research [11].

Mental Health Knowledge Schedule (MAKS)

This scale was employed to check knowledge regarding mental illnesses, with a special emphasis on stigma. This scale has two parts: part A comprises six items related to knowledge about stigma and mental health, while part B consists of six questions to check the participants’ knowledge of common mental health disorders and their ability to classify them. The scores range from 1, which indicates participant response of ‘strongly disagree’, to 5, which denotes ‘strongly agree’. The MAKS score ranges from 12 to 60, where a higher score suggests greater stigma-related knowledge. The Cronbach’s α for the MAKS scale was observed to be 0.749 in a previous study [12]. 

Reported and Intended Behaviour Scale (RIBS)

The RIBS is a tool to evaluate stigma-related behavioural responses concerning mental illness. This scale has eight items to assess intended and reported behaviour towards individuals with mental disorders. Items 5-8 are assigned scores on a Likert scale with possible scores ranging from one (strongly disagree) to five (strongly agree). Higher total scores are suggestive of a greater degree of expected positive behaviour towards persons with mental ill-health. The RIBS has been demonstrated to have a Cronbach’s α ranging from 0.72 to 0.81 in previous research studies [13].

Statistical analysis

The data analysis was carried out using IBM SPSS software (IBM SPSS Statistics for Windows, IBM Corp., Version 20, Armonk, NY). Continuous variables were expressed using mean ± SD. To test the statistical significance of the difference in the mean values of MICA-4, MAKS and RIBS scores with socio-demographic variables, independent samples t-test for normal data (two-group comparison) and one-way ANOVA (more than two-group comparison) were applied. P-value < 0.05 was deemed statistically significant.

## Results

The participants included 301 pharmacy students comprising 150 PharmD students, 127 BPharm students and 24 MPharmstudents. The majority of the participants were females (81.1%). The mean age of the respondents was 21 ± 1.648 years; 6.3% of the participants had a family member with a psychiatric illness (Table 1).

**Table 1:** Socio-demographic characteristics (n = 301) PharmD: Doctor of Pharmacy, BPharm: Bachelor of Pharmacy, MPharm: Master of Pharmacy

Parameter	Category	Number (n)	Percentage (%)
Age	≤21	191	63.45
	>21	110	36.5
Gender	Males	57	18.9
	Females	244	81.1
Course	PharmD	150	49.8
	BPharm	127	42.2
	MPharm	24	8.0
Year of study	First year	17	5.64
	Second year	74	24.58
	Third year	63	20.93
	Fourth year	60	19.9
	Fifth year	46	15.28
	Sixth year	41	13.62
Family history of psychiatric illness	Yes	19	6.3
	No	282	93.7

The attitude of the students concerning mental illness was assessed using MICA-4 scores, the mean value of which was found to be 44.52 ± 7.662. Scores on MICA-4 range from 16 to 96, where increasing scores denote a more negative attitude. The mean RIBS score was found to be 13.18 ± 4.884 (possible score range of 4-20 with higher scores representing less intended behavioural discrimination). Knowledge and understanding about the stigma associated with mental health illness were assessed using the MAKS score, the mean value of which was 45.3156 ± 7.601 (score range of 12-60 with greater scores indicating more mental health knowledge concerning stigma).

The responses on the MICA-4 scale revealed that among the participants, 45.6% would not inform their friends if they had a mental illness; 58.1% responded that they would be hesitant to divulge their mental illness to their colleagues as they were concerned about being treated differently; 48.5% of respondents found it uncomfortable to have a conversation with individuals with mental illness. More than half of the respondents (59.8%) considered psychiatric patients as dangerous, and nearly 74.7% opined that persons with mental illness need to be secluded from the general public. A small proportion of students (14.6%) reported that they sometimes resorted to using terms such as ‘crazy’ and ‘mad’ to address psychiatric patients whom they encountered at their workplace; 77.5% of the study participants opined that recovery from severe mental illness is possible and that these individuals can go on to have a good quality of life; 93% of participants believed that psychiatry is a respectable medical speciality (Table 2).

**Table 2:** Participant responses regarding attitude towards mental illness on MICA-4 MICA-4: Mental Illness: Clinicians' Attitudes Scale

MICA-4 questions	Agree n (%)	Neutral n (%)	Disagree n (%)
I just learn about mental health when I have to and would not bother reading additional material on it. I just learn about mental health when I have to and would not bother reading additional material on it. I just learn about mental health when I have to, and would not bother reading additional material on it.	95 (31.6)	116 (38.6)	90 (29.9)
People with a severe mental illness can never recover enough to have a good quality of life.	25 (8.3)	91 (30.3)	185 (61.2)
Working in the mental health field is just as respectable as other fields of health and social care.	280 (93)	13 (4.3)	8 (2.6)
If I had a mental illness, I would never admit this to my friends because I would fear being treated differently.	51 (17)	137 (45.5)	113 (37.6)
People with severe mental illness are dangerous more often than not.	70 (23.3)	169 (56.1)	62 (20.6)
Health/social care staff know more about the lives of people treated for a mental illness than do family members or friends.	171 (56.8)	109 (36.2)	21 (7)
If I had a mental illness, I would never admit this to my colleagues for fear of being treated differently.	63 (20.9)	150 (49.8)	88 (29.2)
Being a health/social care professional in the area of mental health is not like being a real health/social care professional.	51 (16.9)	67 (22.3)	183 (60.8)
If a senior colleague instructed me to treat people with mental illness in a disrespectful manner, I would not follow their instructions.	243 (80.7)	32 (10.7)	26 (8.6)
I feel as comfortable talking to a person with a mental illness as I do talking to a person with a physical illness.	155 (51.5)	131 (43.5)	15 (5)
It is important that any health/social care professional supporting a person with a mental illness also ensures that their physical health is assessed.	258 (85.7)	38 (12.6)	5 (1.6)
The public does not need to be protected from people with severe mental illness.	76 (25.2)	141 (46.8)	84 (27.9)
If a person with a mental illness complained of physical illness (such as chest pain) I would attribute it to their mental illness.	46 (15.3)	89 (29.6)	166 (55.2)
General practitioners should not be expected to complete a thorough assessment of people with psychiatric symptoms because they can be referred to a psychiatrist.	88 (29.2)	125 (41.5)	88 (29.2)
I would use the terms ‘crazy’, ‘nutter’, ‘mad’ etc. to describe to colleagues people with mental illness who I have seen in my work.	22 (7.3)	29 (9.6)	250 (83.1)
If a colleague told me they had a mental illness, I would still want to work with them.	222 (73.8)	69 (23)	10 (3.3)

According to the participant responses on MAKS, it was noted that the majority (89.4%) deemed psychotherapy as effective in mental illness; 24.6% of the respondents did not consider medication as an effective treatment modality for mental illness; 70.1% of the student pharmacists reported that were capable of assisting a friend with mental ill-health in seeking professional help. A vast majority of students recognised schizophrenia (82.4%), bipolar disorder (90.4%) and depression (87.1%) as psychiatric conditions. However, 41.5% of them did not adjudge drug addiction to be a mental illness (Table 3).

**Table 3:** Participant responses regarding knowledge about mental illness on MAKS MAKS: Mental Health Knowledge Schedule

MAKS questions	Agree n (%)	Neutral n (%)	Disagree n (%)	Do Not Know n (%)
Q1 Most people with mental health problems want to have paid employment.	198 (65.7)	79 (26.2)	6 (2)	18 (6)
Q2 If a friend had a mental health problem, I know what advice to give them to get professional help.	211 (70.1)	42 (14)	15 (5)	33 (11)
Q3 Medication can be an effective treatment for people with mental health problems.	227 (75.4)	44 (14.6)	18 (6)	12 (4)
Q4 Psychotherapy can be an effective treatment for people with mental health problems.	269 (89.4)	18 (6)	8 (2.7)	6 (2)
Q5 People with severe mental health problems can fully recover.	189 (62.8)	63 (20.9)	16 (5.3)	33 (11)
Q6 Most people with mental health problems go to a healthcare professional to get help.	150 (49.9)	56 (18)	77 (25.6)	18 (6)
Whether the following conditions are labelled to be a mental illness?				
Q7 Depression	262 (87.1)	24 (8)	10 (3.3)	5 (1.7)
Q8 Stress	137 (45.5)	72 (23.9)	67 (22.3)	25 (8.3)
Q9 Schizophrenia	248 (82.4)	23 (7.6)	10 (3.4)	20 (6.6)
Q10 Bipolar disorder	272 (90.4)	17 (5.6)	7 (2.4)	5 (1.7)
Q11 Drug addiction	176 (58.5)	59 (19.6)	46 (15.2)	20 (6.6)
Q12 Grief	129 (42.9)	79 (26.2)	64 (21.2)	29 (9.6)

**Figure 1:**
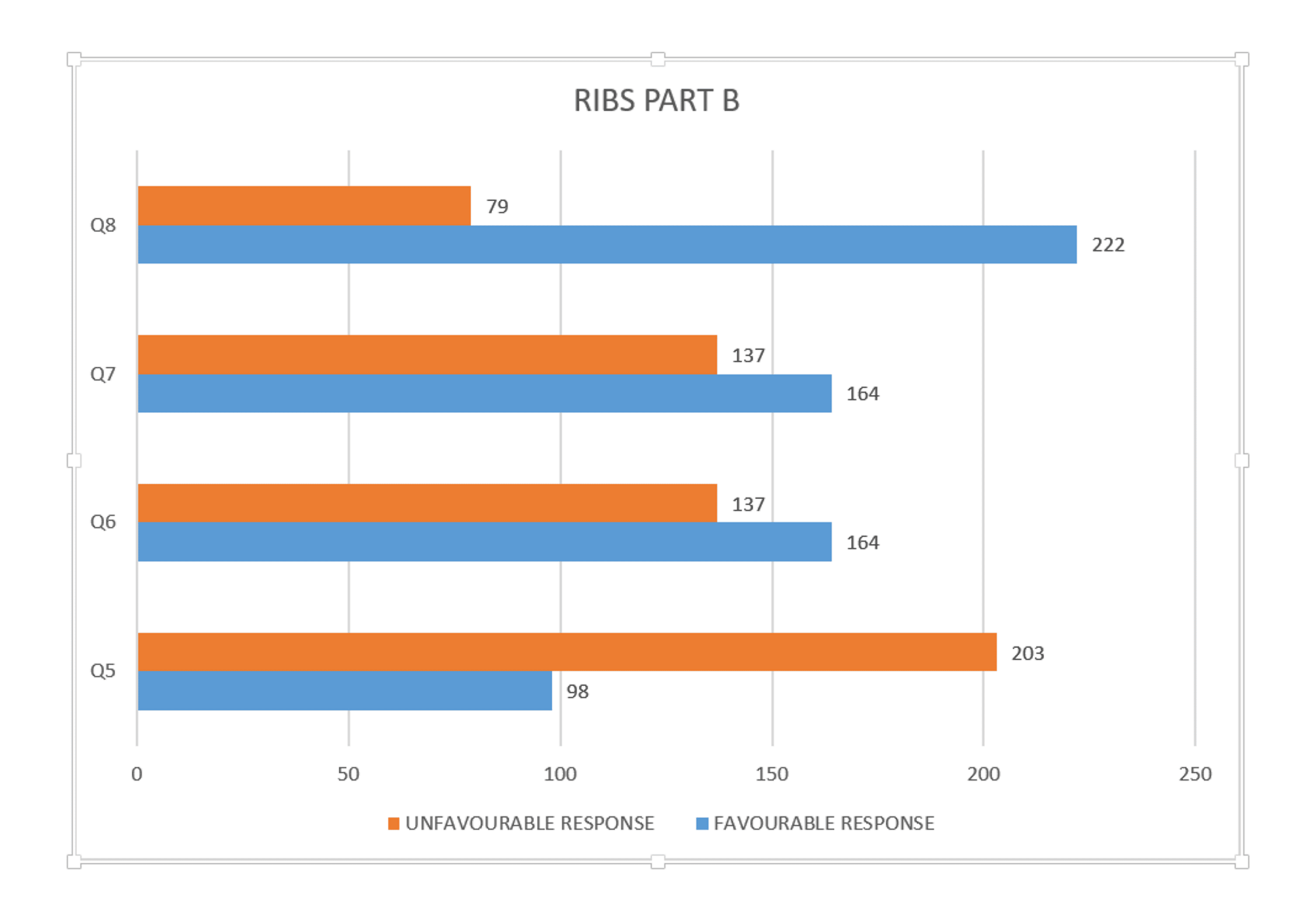
Intended behaviour on RIBS (responses to questions 5-8) RIBS: Reported and Intended Behaviour Scale

The responses to RIBS questions 1 to 4 demonstrated that the majority of the respondents (81.7%) did not have any prior contact with individuals who had mental illnesses. Three-fourths of the sample had neither a colleague nor a close friend with mental illness; 18.9% of the students reported being acquainted with a neighbour who had a mental illness. Responses regarding intended behaviour showed that though 67.5% of the participants were unwilling to live with someone with a psychiatric condition in future, 73.8% did not mind continuing their relationship with a friend who had a mental illness (Figure 1). Questions 1-8 are presented in Supplementary Materials 1-2.

Male participants in the study were noted to have significantly higher MICA scores than females, indicating a more negative attitude toward mental illness (p = 0.001). A significantly lower MICA-4 score was noted among the students enrolled in the PharmD course, indicating a more favourable attitude towards mental illness (p = 0.01) (Table 4).

**Table 4:** Association between MICA scores and socio-demographic variables Independent samples t-test/ANOVA was used to find the p-values *p < 0.05 is considered statistically significant. BPharm: Bachelor of Pharmacy, MPharm: Master of Pharmacy, PharmD: Doctor of Pharmacy

Parameter	Category	Mean (SD)	t	F	p
Age	≤21	44.28 (7.724)	0.722		0.471
	>21	44.95 (7.570)			
Gender	Male	47.58 (8.454)	3.401		0.001*
	Female	43.81 (7.301)			
Course	PharmD	42.89 (7.524)		7.148	0.001*
	BPharm	46.22 (7.540)			
	MPharm	45.79 (7.241)			
Year of study	First year	47.65 (4.716)		1.237	0.292
	Second year	44.23 (7.601)			
	Third year	43.60 (7.136)			
	Fourth year	45.72 (7.894)			
	Fifth year	43.48 (8.153)			
	Sixth year	44.61 (8.444)			

BPharm students exhibited significantly lesser RIBS scores (11.7 ± 5.110) as compared to the students in the other two programs, indicating less desirable intended behaviours (p = <0.001). First-year students had the least RIBS scores (p = 0.004) as compared to the students in the second to sixth years of study. A significantly higher RIBS score was observed in students aged above 21 years (p = 0.009) (Table 5).

**Table 5:** Association between RIBS scores and socio-demographic variables Independent samples t-test/ANOVA was used to find the p-values. *p < 0.05 is considered statistically significant. BPharm: Bachelor of Pharmacy, MPharm: Master of Pharmacy, PharmD: Doctor of Pharmacy

Parameter	Category	Mean (SD)	t	F	p
Age	≤21	12.63 (5.089)	2.620		0.009*
	>21	14.15 (4.364)			
Gender	Male	13.53 (4.302)	0.589		0.556
	Female	13.10 (5.015)			
Course	PharmD	14.29 (4.356)		10.784	<0.001*
	BPharm	11.70 (5.110)			
	MPharm	14.13 (4.919)			
Year of study	First year	10.59 (4.836)		3.6	0.004*
	Second year	11.73 (5.465)			
	Third year	14.27 (4.293)			
	Fourth year	13.33 (5.038)			
	Fifth year	13.89 (3.985)			
	Sixth year	14.20 (4.573)			

A family history of psychiatric illness did not significantly influence the scores obtained on the three scales. The mean score on MAKS did not show a significant association with the sociodemographic variables.

## Discussion

We believe that this is the first Indian study that has tried to evaluate the knowledge, attitude and behavioural responses towards people with mental illness among pharmacy students. The study results are in keeping with various studies across the globe, which indicate that negative beliefs and unfavourable attitudes towards mental illness exist among practitioners as well as students training in healthcare.

In our study, 59.8% of the respondents considered mentally ill individuals as potentially ‘dangerous’ to others. Similar findings were noted in a study conducted among pharmacy students in Saudi Arabia [14]; 74.7% of our subjects thought that those with mental illnesses need to be secluded from mainstream society. This statement indirectly points to the notion that mentally ill patients need to be punished in concern of public safety. Another notable finding is that 67.5% of the subjects expressed unwillingness to live with a person with a psychiatric condition in future. A study conducted among pharmacy students in eight universities across six countries noted that unpredictability and perceived dangerousness of mentally ill individuals were important factors associated with the desire for increased social distancing [15].

A small fraction of students revealed that they tended to use terms like ‘crazy’, ‘nutter’ and ‘mad’ to refer to psychiatric patients, which points towards the stigma concerning people with mental illness. Link et al. conceptualise stigma as comprising four major interrelated components. These include labelling differences, stereotyping, separating, loss of societal status and discrimination [16]. Social selection of human differences, which are then labelled, is usually followed by associating them with attributes or stereotypes that are devalued in the society. Literature shows that the process of labelling and stereotyping typically are ‘automatic’, implying that they tend to operate preconsciously and often lead to split-second judgements. Unless a conscious effort is made to rectify them, this culturally influenced categorisation of individuals will tend to continue. The stigmatised individual tends to be thought of as fundamentally distinct from other members of society who do not carry these labels. This then becomes the basis for excluding them from society and discriminating against them [17].

A notable proportion of students revealed that if they were diagnosed with a psychiatric illness in future, they would be hesitant to disclose this to their friends (45.6%) and colleagues (58.1%) as they feared being treated differently. This hints at the existence of self-stigma or perceived stigma among the students, which refers to the realisation that the public, in general, harbours prejudice and will discriminate against individuals who have mental illnesses. Evidence from research indicates that higher anticipated self-stigma is associated with greater reluctance to seek help [18]. A study conducted among the Belgian population who were presented with vignettes depicting depression or schizophrenia found that anticipated self-stigma was associated with considering that seeking help from a psychiatrist or general practitioner was less important [19]. According to The Stage Model of Self Stigma’ proposed by Corrigan and Rao, the process of internalisation of public stigma occurs through successive stages. These include awareness that public stigma exists about a particular condition, a personal agreement that these negative stereotypes are true and application of these negative public stereotypes to oneself. The authors also propose various methods of challenging self-stigma, which include selective disclosure about the illness to a group of peers and indiscriminate disclosure with broadcasting of one’s experiences with mental illness, which may bring about a sense of power over the illness and the accompanying stigma [20].

Our study findings indicated that female students scored better on the MICA-4 scale, which highlights a greater extent of favourable attitude concerning mental illness among females in comparison to their male counterparts. This was in concurrence with a previous study involving pharmacy students in Yemen [21]. Another observation of our study was that PharmD students had better scores on MICA-4 and RIBS scales, indicating positive attitudes and behaviour towards persons with mental illness as compared to the other two groups, whereas BPharm undergraduates had less favourable scores. This finding can be attributed to more opportunities for clinical exposure in the PharmD training program and the dearth of this aspect in the curriculum of the other two courses. The first-year students of all three programs had less favourable reported and intended behaviour towards the mentally ill than their seniors. This also highlights the fact that training and education regarding mental illnesses as part of the academic curriculum can bring about positive changes in the behaviour of students. A study by Desai et al. among medical undergraduate students compared the attitudes of the students before and after their first psychiatry clinical posting. The authors noted that the outlook towards the subject of psychiatry, as well as the attitude concerning mental illness among the students, became more positive at the end of the clinical postings [22].

Besides mental health stigma, stigma towards psychotropic medication was also noted among the participants. The majority of the participants deemed psychotherapy as a useful treatment modality for psychiatric disorders, while a quarter of the participants did not consider pharmacological treatments as effective. Davis et al. conducted a study among 501 students enrolled in a PharmD program and observed that most of the participants preferred psychological interventions as a stand-alone treatment (42%) or a combination of psychological treatment with medications (40%) [23]. Similar findings are mirrored in our study. Stigma towards psychotropic medications is likely to negatively affect the student pharmacists’ patient counselling regarding the need and utility of medications in treating psychiatric conditions, which can contribute to medication non-adherence among the patients [24]; 41.5% of the students fail to recognise substance dependence as a mental disorder, which may lead to misguidance on the part of the future pharmacist and lead to a delay in seeking timely medical help for the management of substance-use disorders. However, it is noteworthy that a considerable number of respondents were aware that depression, schizophrenia and bipolar disorder are major psychiatric conditions.

The study findings have several implications. The existence of stigmatising beliefs, unfavourable attitudes and behavioural tendencies among future pharmacists about mental illness is a major roadblock to effective mental health service delivery. A therapeutic alliance formed between the patient and the pharmacist based on being able to have an understanding of the struggles and necessities of the patients on these medications is a vital component of medication counselling. Providing opportunities to enhance contact and engagement with people with mental disorders may be an important step in improving the students’ attitudes during pharmacy training at an undergraduate level. A pharmacy curriculum where modules that are focused on providing theoretical knowledge about mental disorders are supplemented with contact-based methods which promote social contact with individuals with mental illness to address stigma among student pharmacists is the need of the hour.

Limitations

1) Single-centred study - This study included subjects from a single tertiary care centre in South India. The use of a cross-sectional study design with convenient sampling requires caution when generalising the results. This approach may not fully represent the broader population, as it limits the diversity and randomness of the sample.

2) Gender bias - The study included a majority of female participants (81.1%). The findings may reflect a particular gender perspective. This imbalance might have overlooked the insights from other genders.

3) Self-report bias - Given that the questionnaire addresses a sensitive topic, such as mental disorder, participants may have been hesitant to provide truthful responses due to fear of being stereotyped or judged. This social desirability bias could have influenced the accuracy of the data collected.

4) Respondent fatigue - The survey length may have led to respondent fatigue as three scales were employed in assessing the outcome of the study.

## Conclusions

This study highlights the existence of stigmatising perceptions regarding individuals with mental illness among student pharmacists. Stigmatising attitudes and behaviours have the potential to hinder help-seeking among patients, affect the patient-pharmacist relationship and lead to suboptimal treatment outcomes. Interventions and modules to address stigma towards mental illness, as well as psychotropic medications, need to be incorporated into the pharmacy curriculum in the country.

## Appendices

Questionnaire

Sociodemographic Details

1) Age

2) Gender

3) Course

4) Year of study

5) Family history of psychiatric illness

**Table 6:** Reported and Intended Behaviour Scale: Part A RIBS: Reported and Intended Behaviour Scale

RIBS	Yes	No	Do not know
Q1 Are you currently living with or have you ever lived with someone with a mental health problem?			
Q2 Are you currently working with or have you ever worked with someone with a mental health problem?			
Q3 Do you currently have or have you ever had a neighbour with a mental health problem?			
Q4 Do you currently have or have you ever had a close friend with a mental health problem?			

**Table 7:** Reported and Intended Behaviour Scale: Part B RIBS: Reported and Intended Behaviour Scale

RIBS	Agree strongly	Agree slightly	Neither agree nor disagree	Disagree slightly	Disagree strongly	Do not know
Q5 In the future, I would be willing to live with someone with a mental health problem.						
Q6 In the future, I would be willing to work with someone with a mental health problem.						
Q7 In the future, I would be willing to live nearby to someone with a mental health problem.						
Q8 In the future, I would be willing to continue a relationship with a friend who developed a mental health problem.						
